# HIV viral suppression and risk of viral rebound in patients on antiretroviral therapy: a two- year retrospective cohort study in Northern Tanzania

**DOI:** 10.1186/s12879-024-09161-y

**Published:** 2024-04-11

**Authors:** Monica S Kahabuka, Yimtubezinash Woldeamanuel, Peter M. Mbelele, Emmanuel A. Mpolya, Stellah G. Mpagama, Jonas P. Kessy, Tsegahun Manyazewal

**Affiliations:** 1 College of Health Sciences, Center for Innovative Drug Development and Therapeutic Trials for Africa (CDT-Africa), Addis Ababa University, Addis Ababa, Ethiopia; 2 Kibong’oto National Infectious Diseases Hospital, Kilimanjaro, Tanzania; 3 Mawenzi Regional Referral Hospital, Kilimanjaro, Tanzania; 4 Department of Global Health and Biomedical Sciences, School of Life Sciences and Bioengineering, Nelson Mandela African Institution of Science and Technology, Arusha, Tanzania; 5 Institute for Health Metrics and Evaluation, Population Health Building/Hans Rosling Center, University of Washington, 3980 15th Avenue, 98195 Seattle, WA USA

**Keywords:** Undetectable viral load, Viral suppression, Viral rebound

## Abstract

**Background:**

The world is moving towards the third target of the Joint United Nations Programme on HIV/AIDS to ensure most people receiving antiretroviral therapy (ART) are virologically suppressed. Little is known about viral suppression at an undetectable level and the risk of viral rebound phenomenon in sub-Saharan Africa which covers 67% of the global HIV burden.This study aimed to investigate the proportion of viral suppression at an undetectable level and the risk of viral rebound among people living with HIV receiving ART in northern Tanzania.

**Methodology:**

A hospital based-retrospective study recruited people living with HIV who were on ART for at least two years at Kibong’oto Infectious Disease Hospital and Mawenzi Regional Referral Hospital in Kilimanjaro Region, Tanzania. Participants’ two-year plasma HIV were captured at months 6, 12, and 24 of ART. Undetectable viral load was defined by plasma HIV of viral load (VL) less than 20copies/ml and viral rebound (VR) was considered to anyone having VL of more than 50 copies/ml after having history of undetectable level of the VL less than 20copies/ml. A multivariable log-binomial generalized linear model was used to determine factors for undetectable VL and viral VR.

**Results:**

Among 416 PLHIV recruited, 226 (54.3%) were female. The mean (standard deviation) age was 43.7 (13.3) years. The overall proportion of undetectable VL was 68% (95% CI: 63.3–72.3) and 40.0% had viral rebound (95% CI: 34.7–45.6). Participants who had at least 3 clinic visits were 1.3 times more likely to have undetectable VL compared to those who had 1 to 2 clinic visits in a year (*p* = 0.029). Similarly, participants with many clinical visits ( > = 3 visits) per year were less likely to have VR compared to those with fewer visits ( = 2 visits) [adjusted relative risk (aRR) = 0.64; 95% CI: 0.44–0.93].

**Conclusion:**

Participants who had fewer clinic visits per year(ART refills) were less likely to achieve viral suppression and more likely to experience viral rebound. Enhanced health education and close follow-up of PLHIV on antiretroviral therapy are crucial to reinforce adherence and maintain an undetectable viral load.

## Background

Human Immunodeficiency Virus (HIV) and its acquired immunodeficiency syndrome (AIDS) is still a public health concern worldwide. So far, HIV has infected 79.3 million people and caused 36.3 million death worldwide, including 0.65 million people who died in 2021 [1]. The Joint United Nations Programme on HIV/AIDS (UNAIDS) pathway to end the HIV epidemic by 2030 led to the set up of an ambitious target to ensure that 95% of all people living with HIV (PLHIV) know their HIV status, 95% of them receive sustained antiretroviral therapy (ART), and 95% of all people receiving ART achieve viral suppression by 2025 [2–4]. Despite these strategies to achieve these targets, the 2022 UNAIDS global report shows that 4000 people become newly infected with HIV every day [5]. HIV/AIDS is an incurable disease, but its progression to severe form can be controlled by using ART. ART medicines suppress viral replication which maintain the HIV plasma viral load to undetectable level while restoring the impaired immunity [6]. This reduces mortality and morbidity consequently improving people’s quality of life.

Ending HIV/AIDS disease require implementation strategies not only for breaking transmission cycle such as early detection, inititiation of ART to restore lost immunity, monitoring treatment response to track viral undetectability and viral rebounds but also strengthening health education to enhance adherence to care, among other inititiave [7]. Undetectable (U) equals Untransmittable (U) colloquial has been a focus for attaining and surpassing both the 90-90-90 and 95-95-95 targets (95/98/97). Undetectability of HIV depends on the threshold below which the HIV viral load (HVL) cannot be detected by the real-time Polymerase Chain Reaction (RT-qPCR) assays [8–10]. This HVL detection threshold by RT-qPCR varies from the previous 50 copies/ml to lower level of 20copies /ml [11].

Undetectable HVL does not mean HIV/AIDS cure rather it escapes killing by host immunity and ART medicines through establishing dormancy in reservoirs cells and tissues like lymphoid tissues [12, 13]. When ART is stopped, HIV reservoirs replicate and increase in number, the phenomenon called *viral rebound/ recrudescence*. To avoid viral rebound after attaining undetectable HVL, health education around the U = U, retention to care and adherence to ART is of paramount importance [8, 9]. Previous studies have proposed a substantial proportion of post-treatment people who do not experience a rebound after stopping ARV where others maintain undetectable without ART [13, 14]. Nonetheless, ART remains to be the cornerstone of HIV so far [12–15].

Global estimate report has showed that 79% of adult PLHIV achieve viral suppression (viral load < 1000 copies per mL) after 1-year of ART [16]. Particulalry in low and middle income countries, some individuals living with HIV do not attain sustained viral suppression. For examples, estimated viral suppression is around 50% in Cameroon and 80% in Namibia whereas it is estimated to be 60% in Tanzania [5]. Nonetheless, viral rebound is now increasingly reported among people who had prior history of viral suppression. A study in Zimbabwe reported that the rates of rebound are higher than viral suppression [17]. However, there is paucity of data on viral rebound and the risk for rebound in most LMIC such as Tanzania. This information is key to guide the design of the public health and clinical interventions for sustaining undetectable HVL, breaking transmission cycle and improving the quality of life.Thus this study aimed to investigate the proportion and predictors of viral suppression at an undetectable level and the risk of viral rebound in PLHIV on antiretroviral therapy in northern Tanzania. By identifying factors contributing to success, the research offers valuable insights for refining programmatic activities related to the follow-up of PLHIV and enabling healthcare practitioners and policymakers to tailor interventions that align with the specific needs of populations.

## Methods

### Study design and participants

This was a hospital based-retrospective study of people living with HIV/AIDS (PLHIV) who were enrolled in HIV care between 2016 and 2020. Each patient’s virological response of 6,12 and 24months were collected. The latest VL results taken within three months at the time of study enrolment was also collected. These PLHIV were attending care and treatment clinics (CTCs) at different intervals ranging from monthly to every 6 months per year and they received either of the following regimen: first line-based regimen such as Tenofovir + Lamivudine + Dolutegravir (TDF + 3TC + DTG); Abacavir + Lamivudine + Dolutegravir (ABC + 3TC + DTG); Tenofovir + Emtricitabine + Efavirenz (TDF + FTC + EFV) and Tenofovir + Lamivudine + Efavirenz (TDF + 3TC + EFV) and the second line-based regimen used by participants are Abacavir + Lamivudine + Lopinavir/ritonavir (ABC + 3TC + LPV/r) and Tenofovir + Emtricitabine + Atazanavir/ritonavir (TDF + FTC + ATV/r).

Data collection was done in Kilimanjaro Region, Northern Tanzania from 27th June to 24th July 2022. The data collection was done specifically from the Care and Treatment Center (CTCs) located within the Kibong’oto Infectious Diseases Hospital (KIDH) and the Mawenzi Regional referral hospital (MRRH). KIDH is the super-specialized national hospital for clinical management of HIV and TB. The Centre has a well-organized research infrastructure and a well-kept HIV database. It provides HIV care and treatment services for about 846 clients. MRRH is a high-volume facility serving 3795 clients in HIV care and treatment unit who come from different areas in the Kilimanjaro region.All these individuals are currently receiving ongoing HIV care and treatment services and are regularly monitored at both facilities. Both facilities have diagnostic equipment such as HIV viral load machines necessary for HIV clients’ follow-up as opposed to other facilities which need to take samples to other facilities for VL tests.

PLHIV were included in the study if aged ≥ 5years at the entry of care, actively attending CTC at the study sites, on ART for at least 24 months and had viral test results taken within 3 months at the time of enrollment to the study. Those without at least one follow-up viral load results and transferred in or out were excluded from this study.

The sample size for this study was estimated using an expected prevalence of undetected viral load of at least 50% in PLHIV on ART. With a significant level of 5%, a 0.5 expected proportion in population, 0.05 absolute error of precision, and assuming that 95% of PLHIV would have at least two HIV viral load test results, a minimum of 404 participants were required. Ultimately, a total of 416 PLHIV were enrolled.

A **s**ystematic random sampling technique was used which aimed at having 1:1 participants in both sites. In brief, the lists of eligible participants at both sites were prepared, then sampling started by selecting a participant on the list at random every second participant (at KIDH) and third participant (at MRRH) were included. This procedure was repeated until the required sample size was obtained at each site. Interval for selection was calculated using the formular, K = *N/n*, where; *N* represents total population size i.e., 693 in MRRH and 389 in KIDH. *n* is the sample size (202 each site, making a total sample size of 404, divided equally between the two sites,).We ended up getting 204 partcipants from KIDH and 212 from MMRH.

### Data collection

The clinical report form (CRF) was used to collect participants’ data. Data collected included socio-demographics and the clinical information such as age, sex, baseline and current CD4 count, type of ARV currently used, history of ART switch,baseline and current WHO HIV clinical stage, baseline and current nutritional status (BMI), history of having treatment supporter who visits for drug-refill on behalf of the client(TS visit), marital status, clinic visit interval for the past one year, adherence status(Good adherence was considered to anyone who attended visits without missing or missed less than 2 visits and not missed any dose or missed only 2 or less doses, and plasma HIV viral loads). All data were retrieved from CTC electronic or paper-based databases, patient’s medical records and charts. The HIV plasma viral load were collected at month 6, 12, 24 and the latest VL. All data collected were entered manually in a spreadsheet and cleaned before statistical analysis.

A cleaned dataset was analyzed using Stata version 15.1 (Stata Corp, College Station, TX). Descriptive statistics were summarized using mean (standard deviation) and median (Inter quartile range) for continuous variables, and categorical variables were summarized by frequency and proportions and presented by table and narrations. A proportion of viral suppression was computed as the number of PLHIV who sustained < 1000 copies/ml, undetectable (< 20copies/ml), and unsuppressed (> 1000copies/ml) over all the study participants included in the analysis during the follow-up time. The pairwise deletion was used to handle missing data i.e., participants with some missing data were included in the analysis of other variables with non-missing values.

### Statistical analysis

A chi-squared or Fisher exact test was used to compare the proportion between undetectable viral load (computed from the latest participants’ viral load results) and independent variables to provide counts, proportion, and p-values. The multivariable log-binomial generalized linear model with a robust estimator was used to determine the factors associated with undetectable viral load (< 20 copies/ml). The model assumes a binomial distribution for a binary outcome and it satisfies the model diagnostics and stability criteria.This model is a better estimator of relative risks than the logistic regression when the proportion of the outcome exceeds 10%. Crude and adjusted Relative Risk (RR) with their respective 95% confidence interval (CI) were reported to estimate the strength and magnitude of the association. Univariate analyses were performed by fitting each explanatory variable against the response variable. Explanatory variables with p-values of < 0.05 in the univariate analyses and those considered in the literature as a potential confounders, and of clinical significance were included in the development of multivariable analyses. In addition, multicollinearity between exposures was assessed. Statistical significance was considered for a p-value of less than 0.05.

## Results

### Sociodemographic and clinical characteristics of study participants

**Fig. 1:**
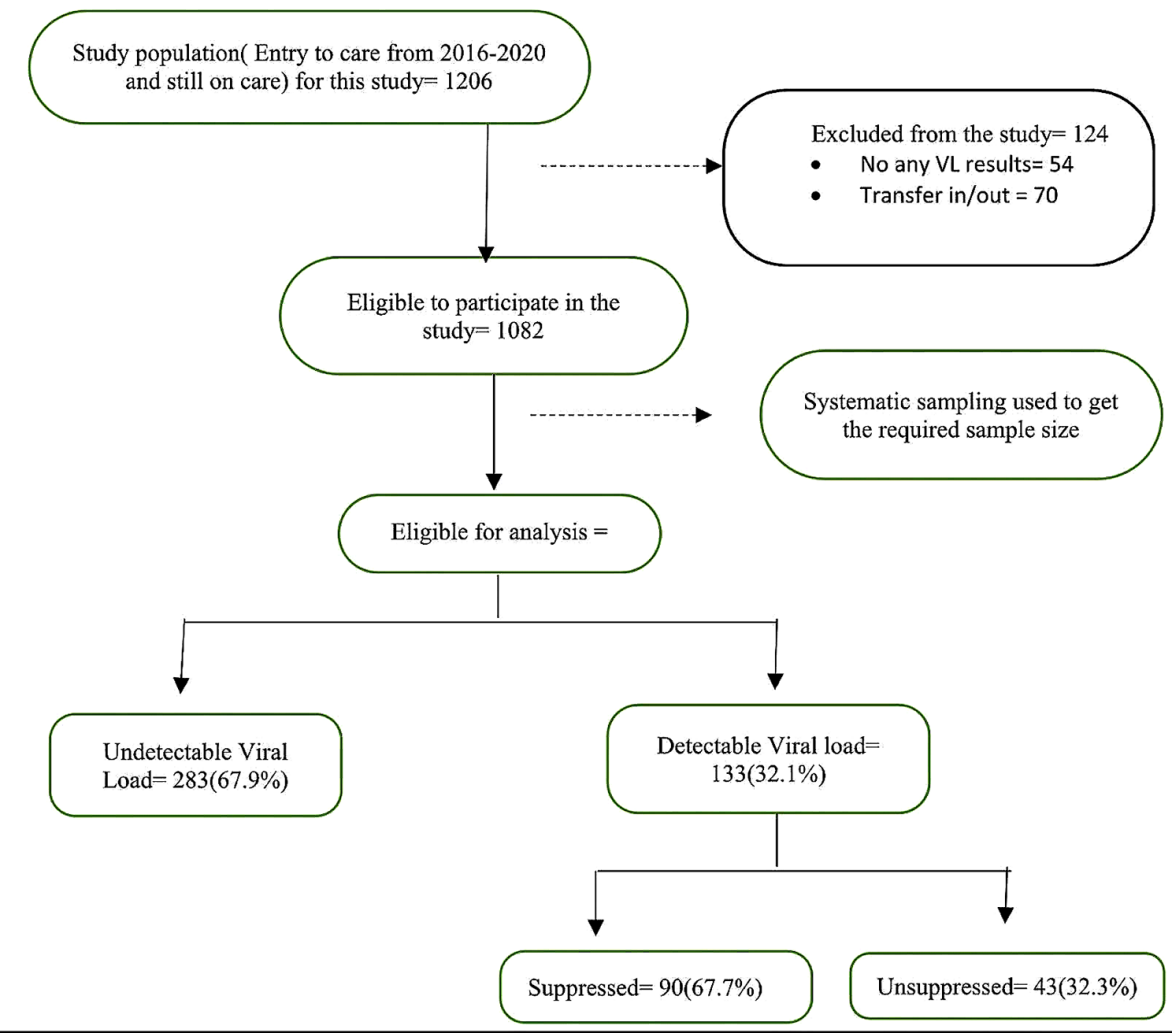
Recruitment process of study participants. *Note* An undetectable viral load, defined as less than 20 copies/mL, was observed in 67.9% of the participants. Suppressed refers to those individuals with viral loads exceeding 20 copies/mL but less than 1000 copies/mL(67.7%). Participants with viral loads above 1000 copies/mL are categorized as unsuppressed(32.3%)

In total, 1206 participants (entry to care from 2016-2020 and still on care ) were screened, of which, 124 (10.3%) were excluded and 416 were eligible for the final analysis. The reasons for exclusion are described in Fig. 1.

Among 416 PLHIV, 226 (54.3%) were female. Population had the mean (SD) age of 43.7 (13.3) years. Detailed characteristcistics of participants are in Table 1. In summary, among 133 PLHIV with detectable viral load, 11 (8.3%) were in WHO clinical stage IV compared to 21 (4.0%) of 283 with undetectable viral load (*p* = 0.002). Similary, 14 (11%) of 133 were underweight compared to 18 (6%) of 283 PLHIV with detectable and undetectable viral load, respectively (*p* = 0.026). In total, 39 (29%) of 133 PLHIV with detectable viral load had 1 to 2 clinic visits compared to only 42 (15%) of 283 PLHIV with undetectable viral load (*p* = 0.002).

### Viral load detection in PLHIV

Among 416 PLHIV, 283 (68.0%, 95% CI: 63.3–72.5) had an overall undetectable HIV plasma viral load at a threshold of < 20copies/ml Among 133 PLHIV with detectable HIV plasma viral load, 72 (54.1%) and 11 (8.3%) had WHO clinical stage III and IV compared to 100 (35.3%) and 21 (4.0%) of 283 PLHIV with undetectable viral load, respectively (*p* = 0.002). Also,Mawenzi Hospital had a higher number of clients with an undetectable viral load at 178 (62.9%, 95%CI: 57.0-68.5) compared to Kibong’oto Hospital(*p* < 0.001).

Among 293 participants aged between 25 and 54 years, 201(71.0%,95%CI: 80.8–89.4) had a higher proportion of undetectable viral load compared to the rest of the age groups (*p* = 0.087, Regarding visit interval,153 PLHIV with three visits or 4–6 visits (182) per year had 113(45.2%) and 128(40.0%) proportions of undetectable viral loads respectively, the proportions of which were higher than those with 1–2 visits (*p* = 0.002,Among 399 participants who reported to have good ART adherence, 275(97.2%,95%CI: 94.5–98.7) of them had a higher proportion of undetectable viral load compared to those with poor adherence (*p* = 0.058). More results are shown in (Table 1).

**Table 1:** Characteristics of participants with detectable and undetectable viral load (*N* = 416)

Characteristics	Total n	Detectable viral load (≥ 20 copies/ml) (*n* = 133)[95% CI]	Undetectable viral load (< 20 copies/ml) (*n* = 283) [95% CI]	p-value
**Age group (in years)**				**0.087** ^**a**^
6–14	20 (4.8)	10 (7.5)	10 (3.5)	
15–24	14 (3.4)	4 (3.0)	10 (3.5)	
25–54	293 (70.4)	92 (69.2)	201 (71.0)	
55+	89 (21.4)	27 (20.3)	62 (22.0)	
*Mean (Standard deviation)*	43.7 (± 13.3)			
**Sex**				**0.081**
Male	190 (45.7)	69 (51.9)	121 (42.8)	
Female	226 (54.3)	64 (48.1)	162 (57.2)	
**Marital status**				**0.655**
Single	85 (20.4)	25 (18.8)	60 (21.2)	
Cohabiting/Married	243 (58.4)	82 (61.7)	161 (56.9)	
Divorced/Separated	88 (21.2)	26 (19.5)	62 (21.9)	
**HIV Stage**				**0.002**
Stage I	92 (22.2)	22(16.5)	70 (24.7)	
Stage II	119 (28.7)	28 (21.1)	91 (32.2)	
Stage III	172 (41.4)	72 (54.1)	100 (35.3)	
Stage IV	32 (7.7)	11 (8.3) [	21 (4.0)	
^*^ **BMI category(kg/m** ^**2**^ **)**				**0.026**
Underweight (< 18.5)	32 (7.7)	14 (10.5)	18 (6.4)	
Normal (18.5-<25)	211 (50.7)	76 (57.1)	134 (47.5)	
Overweight (25-29.99)	93 (22.4)	27 (20.3)	66 (23.4)	
Obesity (≥ 30)	80 (19.2)	16 (12.1)	64 (22.7)	
*Median (Interquartile range)*	23.9 (20.8–28.1)			
**CD4 cell count (cell/mm** ^**3**^ ) *				**0.363**
< 200	48 (13.4)	16 (13.1)	32 (13.5)	
200–350	76 (21.2)	31 (25.4)	45 (19.0)	
> 350	235 (65.4)	75 (61.5)	160 (67.5)	
*Media (Interquartile range)*	448 (280–644)			
**Missing visit**				**0.260**
No	387 (93.0)	121 (91.0)	266 (94.0)	
Yes	29 (7.0)	12 (9.0)	17 (6.0)	
**ART regimen**				**0.214**
First line	401 (96.4)	126 (94.7)	275 (97.2)	
Second line	15 (3.6)	7 (5.3)	8 (2.8)	
**Treatment supporter visit**				**0.651**
No	349 (83.9)	110 (82.7)	235 (84.2)	
Yes	67 (16.1)	23 (17.3)	44 (15.8)	
**ART adherence**				**0.058**
Poor	17 (4.1)	9 (6.8)	8 (2.8)	
Good	399 (95.9)	124 (93.2)	275 (97.2)	
*Visit status				**0.002**
1–2	81 (19.4)	39 (29.3) ]	42 (14.8)	
3	153 (36.8)	40 (30.1)	113 (40.0)	
4–6	182 (43.8)	54 (40.6)	128 (45.2)	
**Facility type**				**< 0.001**
Kibong’oto Hospital	204 (49.0)	99 (74.4)	105 (37.1)	
Mawenzi Hospital	212 (51.0)	34 (25.6)	178 (62.9)	

^Key *Frequency does not tally due to missing values, a Fisher’s exact p−value; visits status is defined as number of visits done per year. E.g.; 1–2 the person, went to the clinic after every 6–12months. *BMI= Current BMI^

### Proportion of viral suppression and undetectable viral loads

**Fig. 2:**
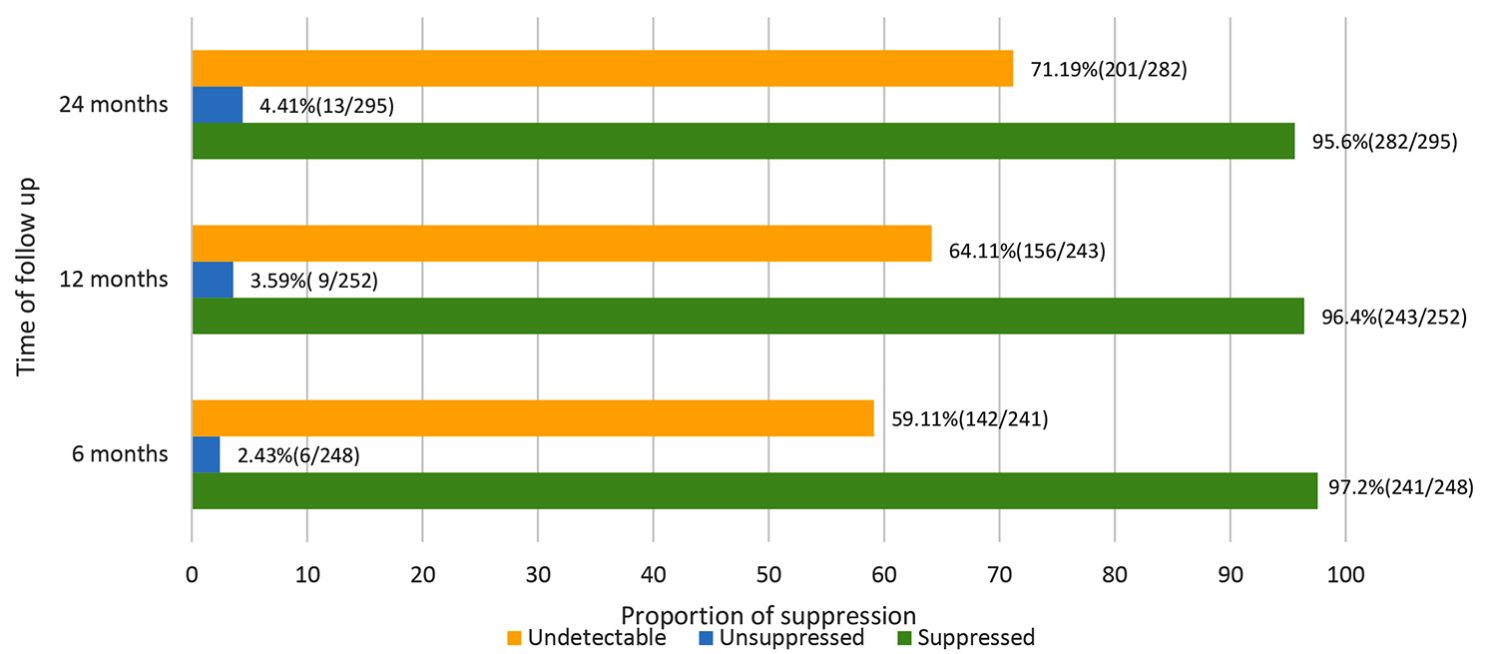
Proportion of viral suppression and undetectable viral loads by time of follow-up among PLHIV on ART in Northern Tanzania

The proportion of viral load suppression (< 1000 copies/ml) exhibits a negligible difference from 6 to 24 months of follow-up, looks stable overtime i.e., 97.2%(241/248, 95%CI: 94.3–98.8), 96.4%(243/252, 95%CI:93.3–98.4) and 95.6%(282/295, 95%CI:92.6–97.6) respectively. On the contrary proportion of undetectable viral load (< 20 copies/ml) increases at every follow-up visit, after six months 59.11%(142/241, 95%CI: 52.9–65.5), twelve months 64.11%(156/243, 95%CI: 58.0-70.2) and twenty-four months 71.19%(201/282, 95%CI: 65.7–76.3) as shown in (Fig. 2).

### HIV plasma viral rebound among PLHIV on ART

**Fig. 3:**
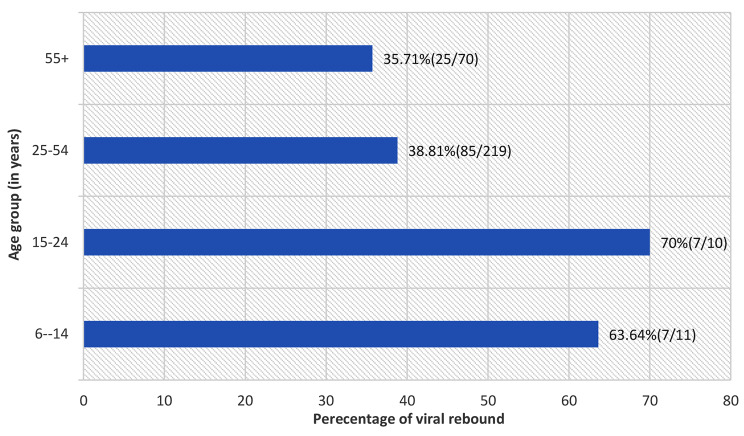
Percentage of viral rebound among PLHIV on ART from 2016 to 2020 in Northern Tanzania stratified by age group (*N* = 124)

40% (124/310, 95%CI: 34.5–45.7) of all PLHIV who had viral suppression experienced a viral rebound. The proportion of viral rebound varied across age groups, whereas PLHIV aged 15–24 had the highest proportion of viral rebound, 7/10(70.0%,95%CI: 34.8–93.3) and those aged 55 years and above had a lower proportion of viral rebound, 25/70 (35.7%, 95%CI: 24.6–48.1) as shown in (Fig. 3).

### Factors associated with undetectable viral load among PLHIV on ART

Obesity is 1.2 times as likely to have undetectable VL compared to PLHIV who had normal nutritional status defined by BMI of 18.5–24.9 kg/m2 (*p* = 0.023). PLHIV who had at least 3 visits to the CTC had at least 1.3 times as likely to have undetectable VL compared to those who had 1 to 2 clinic visits in a year (*p* = 0.029, Table 2). PLHIV with WHO clinical stage III were 0.23 less likely to have undetectable viral load compared to those who had WHO stage I (*p* = 0.003 (Table 2).

**Table 2:** Factors associated with undetectable viral load among PLHIV on ART from 2016 to 2020 in Northern Tanzania (*N* = 416)

Characteristics	Unadjusted	p-value	Adjusted	p-value
	RR (95% CI)		RR (95% CI)	
**Age group (in years)**				
6–14	1.00		1.00	
15–24	1.43 (0.82–2.48)	0.204	1.44 (0.78–2.67)	0.249
25–54	1.37 (0.88–2.14)	0.164	1.33 (0.78–2.26)	0.297
55+	1.39 (0.89–2.21)	0.157	1.33 (0.77–2.31)	0.308
**Sex**				
Male	1.00		1.00	
Female	1.13 (0.98–1.29)	0.086	1.06 (0.93–1.22)	0.379
**Marital status**				
Single	1.00		1.00	
Married/Cohabiting	0.94 (0.79–1.11)	0.449	0.99 (0.82–1.19)	0.910
Divorced/Separated	0.99 (0.82–1.21)	0.985	1.01 (0.81–1.26)	0.908
*Current BMI (kg/m ^**2**^ **)**				
Underweight	0.88 (0.64–1.22)	0.443	0.94 (0.69–1.28)	0.699
Normal	1.00		1.00	
Overweight	1.11 (0.94–1.31)	0.208	1.17 (0.99–1.37)	0.059
Obesity	1.25 (1.08–1.45)	0.003	1.21 (1.03–1.42)	0.023
**HIV Stage**				
Stage I	1.00		1.00	
Stage II	1.01 (0.86–1.17)	0.948	1.00 (0.86–1.17)	0.984
Stage III	0.76 (0.64–0.91)	0.002	0.77 (0.65–0.91)	0.003
Stage IV	0.86 (0.65–1.14)	0.294	0.92 (0.71–1.19)	0.515
**ART regimen**				
First line	1.29 (0.79–2.07)	0.303	0.96 (0.56–1.63)	0.878
Second line	1.00		1.00	
*Visit status				
1–2	1.00		1.00	
3	1.42 (1.13–1.79)	0.003	1.36 (1.08–1.72)	0.009
4–6	1.36 (1.08–1.71)	0.010	1.29 (1.03–1.63)	0.029
**Treatment supporter**				
No	1.00		1.00	
Yes	0.96 (0.79–1.16)	0.661	0.99 (0.81–1.21)	0.913
**ART adherence**				
Poor	1.00		1.00	
Good	1.46 (0.88–2.44)	0.142	1.46 (0.89–2.39)	0.131

^Key RR: Relative risk, 1: Reference.* Visits status is defined as number of visits done per year. E.g.; 1–2 the person, went to the clinic after every 6–12months^

### Factors associated with viral rebound among PLHIV on ART

On crude analysis; participants aged 25–54 years had a 39% lower risk of having viral rebound compared to those aged 6 to 14 years (cRR = 0.61; 95% CI: 0.38–0.98). PLHIV on the first reg/imen had a 0.48 times lower risk of viral rebound compared to those in the second regimen (cRR = 0.52; 95% CI:0.34–0.79).

On adjusted analysis; after adjusting for age group, sex, marital status, CD4, BMI, HIV stage, ART regimen, ART adherence, treatment supporter visits, and visit interval, participants with three-visit intervals had 0.36 times lower risk of viral rebound compared to those with one to two visits (aRR = 0.64; 95% CI: 0.44–0.93). The adjusted model showed that viral rebound did not vary with age and the type of regimen (*p* > 0.05) as shown in Table 3.

**Table 3:** Factors associated with viral load rebound among PLHIV on ART between 2016 to 2020 in Northern Tanzania (*N* = 310)

Characteristics	Unadjusted	p-value	Adjusted	p-value
	RR (95% CI)		RR (95% CI)	
**Age group (in years)**				
6–14	1.00		1.00	
15–24	1.1 (0.60–2.01)	0.757	1.29 (0.61–2.76)	0.505
25–54	0.61 (0.38–0.98)	0.042	0.89 (0.47–1.69)	0.726
55+	0.56 (0.32–0.97)	0.039	0.86 (0.42–1.76)	0.682
**Sex**				
Male	1.00		1.00	
Female	0.86 (0.65–1.12)	0.265	0.86 (0.65–1.13)	0.280
**Marital status**				
Single	1.00		-	
Cohabiting/Married	0.89 (0.65–1.23)	0.481		
Divorced/Separated	0.81 (0.53–1.23)	0.318		
**HIV Stage**				
Stage I	1.00		1.00	
Stage II	0.73 (0.46–1.14)	0.162	0.72 (0.46–1.13)	0.155
Stage III	1.04 (0.73–1.48)	0.813	0.98 (0.69–1.39)	0.907
Stage IV	1.25 (0.79 (1.98)	0.335	1.11 (0.70–1.76)	0.657
*BMI (kg/m ^**2**^ **)**				
Underweight	1.33 (0.84–2.12)	0.218	1.31 (0.81–2.12)	0.264
Normal	1.00			
Overweight	1.28 (0.92–1.79)	0.147	1.22 (0.87–1.72)	0.241
Obesity	1.12 (0.78–1.61)	0.536	1.18 (0.82–1.70)	0.368
**ART regimen**				
First line	0.52 (0.34–0.79)	0.003	0.72 (0.38–1.36)	0.314
Second line	1.00		1.00	
**Treatment supporter visit**				
No	1.00		1.00	
Yes	0.84 (0.57–1.22)	0.360	0.77 (0.51–1.15)	0.205
**ART adherence**				
Poor	1.00		1.00	
Good	0.79 (0.44–1.42)	0.434	0.79 (0.47–1.32)	0.361
*Visit status				
1–2	1.00		1.00	
3	0.59 (0.41–0.85)	0.005	0.64 (0.44–0.93)	0.019
4–6	0.87 (0.62–1.17)	0.307	0.87 (0.63–1.20)	0.397

^Key RR; Relative risk, 1: Reference.* Visits status is defined as number of visits done per year. E.g.; 1–2 the person, went to the clinic after every 6–12months.*BMI= Current BMI^

## Discussion

This study revealed that over two-thirds of PLHIV had an undetectable viral load. Obesity and frequent visits to the CTC emerged as independent predictors of undetectable HVL, whereas those with severe HIV disease at presentation were less likely to achieve an undetectable viral load. Conversely, two in five PLHIV who initially achieved viral suppression experienced viral rebound during the course of treatment. Participants with longer intervals between clinic visits, advanced HIV disease, and younger age were more likely to experience viral rebound within the first two years of ART.

The rate of achieving undetectability appears to rise over time, with over half of the participants reaching this level within the first 6 months after initiating ART. Notably, a substantial majority achieved undetectable levels after 24 months (71.19%). The duration of ART appears to be a significant factor, with a longer duration correlating with a higher likelihood of attaining an undetectable viral load. This trend aligns with findings in the Malawian population, where an increased duration of ART showed a corresponding upward trend in viral suppression [18]. However, it’s important to note a discrepancy in the proportion of undetectable viral load when compared to a study conducted in Croatia, where more than three-quarters of PLHIV reached an undetectable viral load level. The divergence in the threshold of undetectability between the two studies (20 copies/ml in this study and 50 copies/ml in Croatia) may account for this difference [19].

This study also represents the exploration of the connection between frequent clinic visits and achieving an undetectable viral load. The act of regularly visiting the clinic, whether for medication refills or medical consultations, serves as a vital means for PLHIV to engage with care providers, fostering discussions on various aspects of their condition. The health education disseminated at these visits plays a pivotal role in promoting treatment adherence compared to situations where clinic visits are less frequent. Furthermore, the peer support from fellow patients during these visits serves as a source of stress relief and contributes positively to mental health [20, 21]. A comprehensive review spanning nine countries has underscored the superiority of peer support and routine medical care over standard clinic follow-ups in enhancing the outcomes for this population [22]. Supporting our findings, a prior study conducted in a different region in Tanzania highlighted that non-adherence is more prevalent among individuals with longer refill intervals compared to those attending the clinic on a monthly basis [23]. This aligns with our argument that PLHIV with extended clinic visits are less likely to attain undetectable viral load levels, potentially leading to viral rebound, as evidenced in the current study.

Moreover, there exists an inverse relationship between the severity of the disease at presentation and the likelihood of achieving undetectable viral loads. This study illuminates the connection between disease severity and lower viral loads, highlighting that individuals with advanced HIV at presentation often exhibit compromised immunity. Commencing Antiretroviral Therapy (ART) serves to restore immunity, enabling the body to combat the virus. Severe disease presentations, frequently accompanied by serious opportunistic infections, may result in delayed ART initiation for this population to prevent immune response dysregulation [24]. A parallel trend was observed in another Ghanaian study which reported that those with WHO stage I are at higher odds of attaining viral suppression compared to other WHO stages. These two studies employed a similar retrospective design and focused on the same study population (on ART from 2016 to 2020) [25]. This collective evidence underscores the importance of early HIV diagnosis and treatment to avert severe disease, which could otherwise pose challenges in controlling the virus and may lead to adverse outcomes, including mortality. It emphasizes the critical need to educate individuals diagnosed with HIV about the benefits of initiating treatment promptly.

Contrary to expectations, being overweight and obese emerged as independent predictors of undetectable viral load in this study. While prior research has associated weight gain with the use of Dolutegravir (DTG)-based regimens, a significant proportion of PLHIV in the current study were on a DTG-based ART regimen [26–28]. However, due to the retrospective nature of the study, establishing causality was not feasible. The study also indicated that the variation in catchment areas and the clientele served by both sites could explain the differences observed in the number of people attaining undetectable viral load between the two sites. KIDH attracts a substantial number of clients from rural areas with lower education and socio-economic statuses compared to Mawenzi Hospital. Such a population typically requires more time to comprehend the significance of adhering to ART. This finding aligns with a study conducted by Ntokozi and colleagues but contrasts with another study carried out in Malawi [18, 29].

While a substantial number of PLHIV achieved undetectable viral load levels, some encountered challenges in maintaining virological control, experiencing viral rebound at various points during treatment. In this study, over one-third of PLHIV experienced viral rebound over the course of treatment. Notably, a study conducted in Ghana reported a lower prevalence of viral rebound at 21% [30]. The variance could be attributed to the age difference between the studies, as the latter exclusively recruited adult subjects aged 18 years and above, while the current study included participants aged 5 years and above. Adults are generally less prone to experiencing viral rebound compared to children, and issues of adherence may provide an explanation. Many children face challenges in adherence, and even with caregiver assistance, they may be underdosed as their medication is often calculated based on body weight. As they age, dose adjustments become crucial. Several analyses, consistent with the findings of this study, have highlighted the association between younger age and viral rebound [18, 31]. The similarity in results across these studies, conducted in sub-Saharan African countries such as Tanzania, Malawi, and Uganda, may be attributed to the use of routinely collected data and the shared regional context.

Being on the second line of ART increases the vulnerability of PLHIV to experiencing viral rebound. Typically, individuals on the second line have a history of treatment failure, often indicative of suboptimal adherence. A study has highlighted that those with suboptimal adherence to first-line ART are more likely to exhibit suboptimal adherence to second-line ART, with many transitioning to the second line without addressing underlying adherence issues [32]. Moreover, the second-line regimen has been linked to viral load non-suppression, particularly among adolescents who generally exhibit lower adherence compared to adults [33, 34]. This underlines the persistent adherence challenges faced by PLHIV on the second-line regimen. The study emphasizes that transitioning to the second line of ART should not be considered unless adherence issues have been adequately addressed. Even after achieving viral suppression, individuals on the second line may still experience viral rebound, potentially leading to second-line treatment failure—a perilous situation.

It is crucial to note that this study relied on secondary data, and the inherent limitations of such data sources may have impacted data quality. Incompleteness, incorrectness, and missing data in both dependent and independent variables could potentially lead to underestimation or overestimation of associations with undetectable viral load and viral rebound. The use of routinely collected data, primarily designed for programmatic purposes, further poses limitations as certain variables that could provide a more comprehensive understanding of the association between undetectable viral load and viral rebound are not routinely captured in CTC clinics. Examples include HIV status disclosure, substance use, economic status, education level, alcohol intake, and history of smoking. It is essential to acknowledge that the very low viral load threshold utilized in this study differs from the thresholds applied in many other studies, potentially contributing to variations in results. Also the inclusion of both children and adults in the study population presents a notable limitation on the factors. However, this study gives the general obverview of the factors to the HIV population in general.

## Conclusion

The findings regarding the proportion of PLHIV in Kilimanjaro with undetectable viral load levels below twenty copies/ml offer valuable insights. This analysis underscores that less severe disease at presentation and frequent visits to the CTC are predictors of undetectable viral load. Moreover, frequent clinic visits, being on the first-line regimen, and older age have shown to independently contribute to having viral rebound. The study emphasizes the importance of continuous and lifelong follow-up for PLHIV on ART, irrespective of achieving undetectable viral load or viral suppression. This follow-up should be maintained, even if conducted remotely, to ensure ongoing connection with healthcare providers. Educational efforts on the concept of undetectability should be directed towards both clients and care providers. The evidence presented highlights the necessity of consistently reinforcing the importance of adherence during each refill visit, even for those with very low viral loads. Patients need to be reminded that ART is a lifelong treatment, and maintaining adherence is crucial for long-term good health outcomes.

## Data Availability

The datasets generated and/or analysed during the current study are not publicly available due to regulatory restriction i.e. filling of data transfer agreement between author and dataset receiver, however data should be available upon request to the corresponding author.

## Abbreviations

AIDS: Acquired Immunodeficiency Syndrome
ART: Antiretroviral Treatment
ARV: Antiretroviral Virus
BMI: Body Mass Index
CD4: Cluster of Differentiation 4
CTC: Center for Care and Treatment
DTG: Dolutegravir
HIV: Human Immunodeficiency Virus
KIDH: Kibong’oto Infectious Disease Hospital
LMIC: Low- and middle-Income Countries
MRRH: Mawenzi Regional Referral Hospital
PCR: Polymerase Chain Reaction
PLHIV: People Living With HIV
RNA: Ribonucleic Acid
UNAIDS: United Nations Programme on HIV/AIDS
UVL: Undetectable Viral load
VL: Viral Load
VR: Viral Rebound
WHO: World Health Organization
